# Aortoesophageal fistula treated using one-stage total reconstruction: a case report from a high-volume center

**DOI:** 10.1186/s13019-023-02438-4

**Published:** 2023-11-14

**Authors:** Yi-Ping Lee, Kensuke Ozaki, Susumu Oshima, Tomohiro Hirokami

**Affiliations:** 1 Kawasaki Aortic Center, Kawasaki Saiwai Hospital, Kawasaki, Japan; 2 Division of Thoracic and Cardiovascular Surgery, Hualien Tzu Chi Hospital, Buddhist Tzu Chi Medical Foundation, Hualien, Taiwan

**Keywords:** Aortoesophageal fistula, Total arch replacement, Thoracic endovascular aortic repair

## Abstract

**Background:**

Aortoesophageal fistula (AEF) is a rare but typically life-threatening condition. Although several treatment strategies exist, including conservative treatment with intraluminal stent graft and open thoracic aortic replacement, the overall outcome remains poor, ranging from 16 to 39%. Furthermore, esophageal reconstruction methods vary between hospitals. Herein, we report a case of aortoesophageal fistula treated using one-stage total reconstruction.

**Case presentation:**

This case involved a 58-year-old woman who developed acute type A aortic dissection and underwent successful total arch replacement at the other hospital. However, she developed AEF 1 year later and underwent urgent thoracic endovascular aortic repair, which eventually failed. We performed thoracic aortic replacement, total esophagectomy, gastric tube reconstruction, and omental flap in a one-stage operation. The patient was extubated the next day and transferred to the general ward on postoperative day 3. Computed tomography revealed favorable results.

**Conclusions:**

For postoperative AEF, dedicated debridement with reconstruction is more effective than conservative treatment. In an experienced center, post-procedure-related AEF can be easily treated using one-stage reconstruction.

The patient was a 58-year-old woman with no known medical history. She developed a sudden onset of chest pain on November 2, 2020, and an immediate chest computed tomography (CT) scan revealed acute type A aortic dissection (Fig. 1). She underwent urgent total arch replacement on the same day. The postoperative course was uneventful, and the patient was discharged on postoperative day 23. However, on November 22, 2021 (1 year after the operation), she began to report intermittent high-spiking fever, and a follow-up CT revealed air pocket accumulation around the graft anastomotic site (Fig. 2). Blood culture revealed *Streptococcus sanguinis*. Consequently, she was admitted, and antibiotics were started. She underwent thoracic endovascular aortic repair (TEVAR) on November 25, 2021. During the admission course, her vital signs remained stable, and a follow-up CT scan 1 month later revealed improvement in periaortic inflammation without air pocket formation (Fig. 3). Because her condition remained stable, she was discharged after a 6-week course of parental antibiotics.

**Fig. 1:**
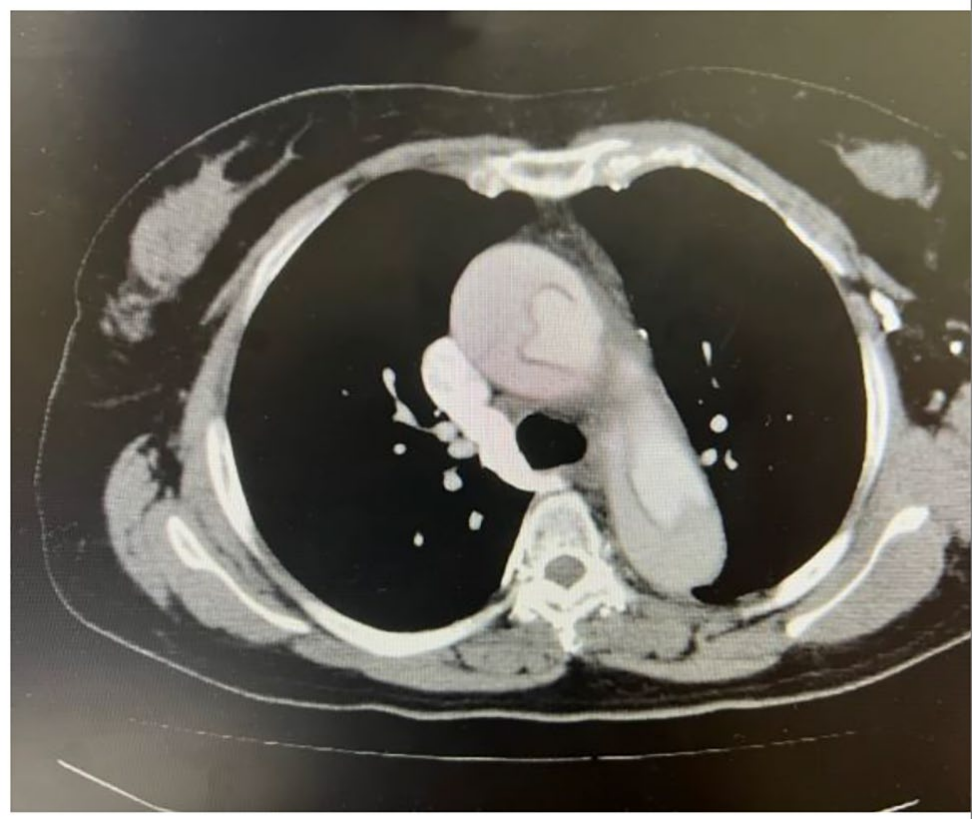
Acute type A aortic dissection

**Fig. 2:**
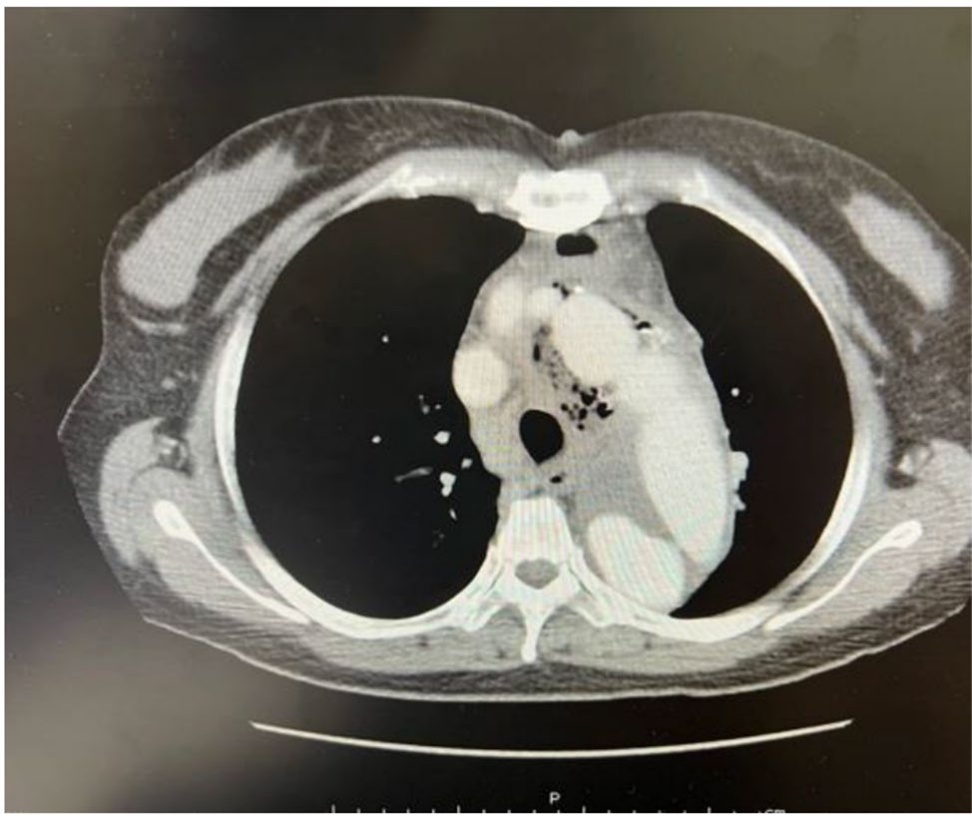
Air pocket formation around aorta

**Fig. 3:**
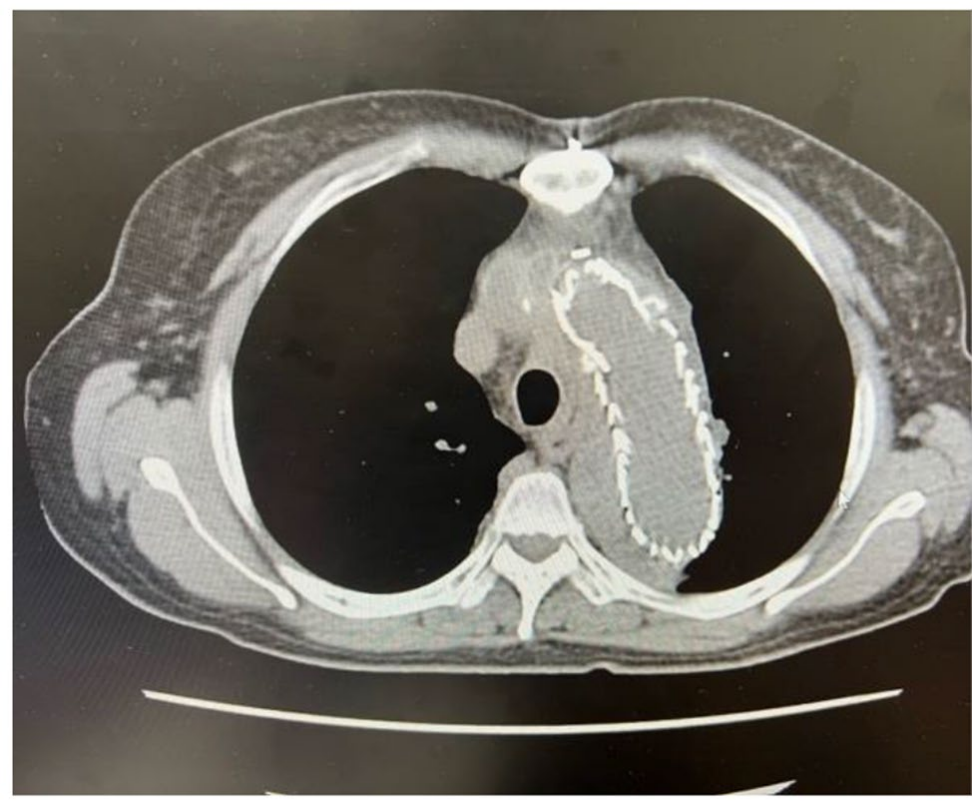
After TEVAR stent

However, the patient reported another episode of high-spiking fever 4 months after discharge. A CT scan on May 31, 2022, revealed little air accumulation around the previous TEVAR stent. The level of the inflammatory marker, C-reactive protein (CRP), was 26.22 mg/L. Owing to progressing sepsis, parental antibiotics were resumed, and she was transferred to our hospital for further management. We maintained the antibiotic treatment and arranged serial examination. Esophagogastroduodenoscopy, which was performed to check the AEF site, revealed an erosion site at 25 cm below the incisor. (See at Fig. 5.) PET-MRI scan was arranged as well to determine the range of graft infection, which showed the extent of infection was localized at the aortoesphageal fistula site. (See at Fig. 6.) During this round of admission, her vital signs were stable under antibiotic therapy, and we arranged total reconstruction surgery after a 14-day antibiotic course. Because we preferred to remove the TEVAR stent graft and repair aortoesophageal fistula, we chose lateral thoracotomy for better surgical field exposure.

**Fig. 4:**
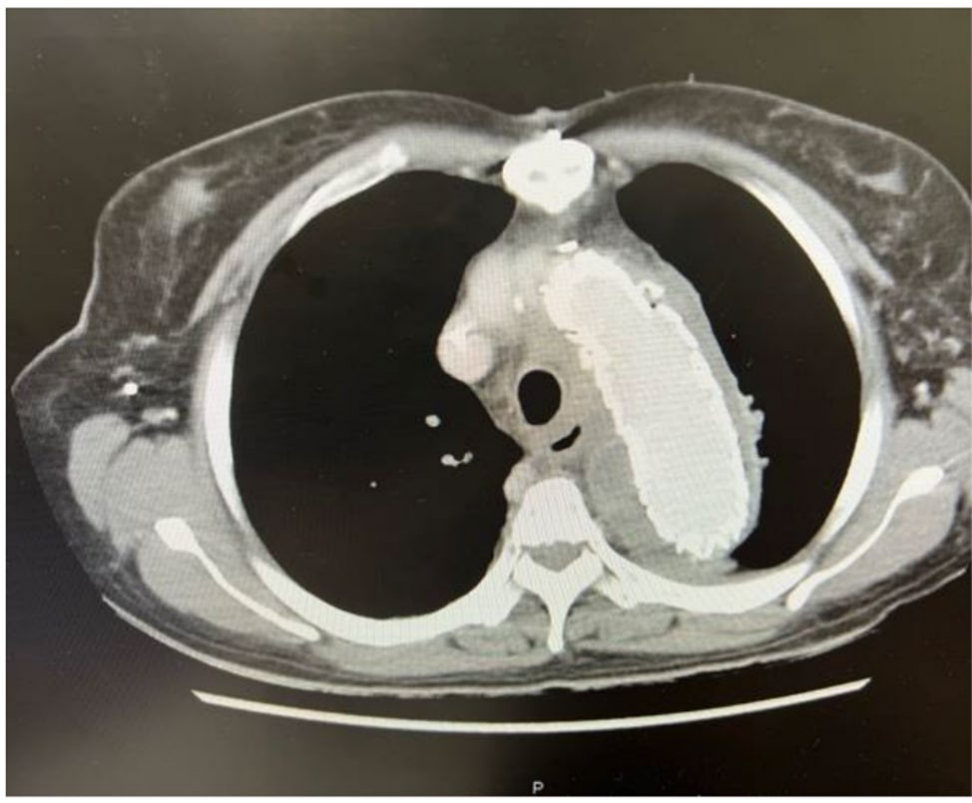
New air formation around TEVAR stent

**Fig. 5:**
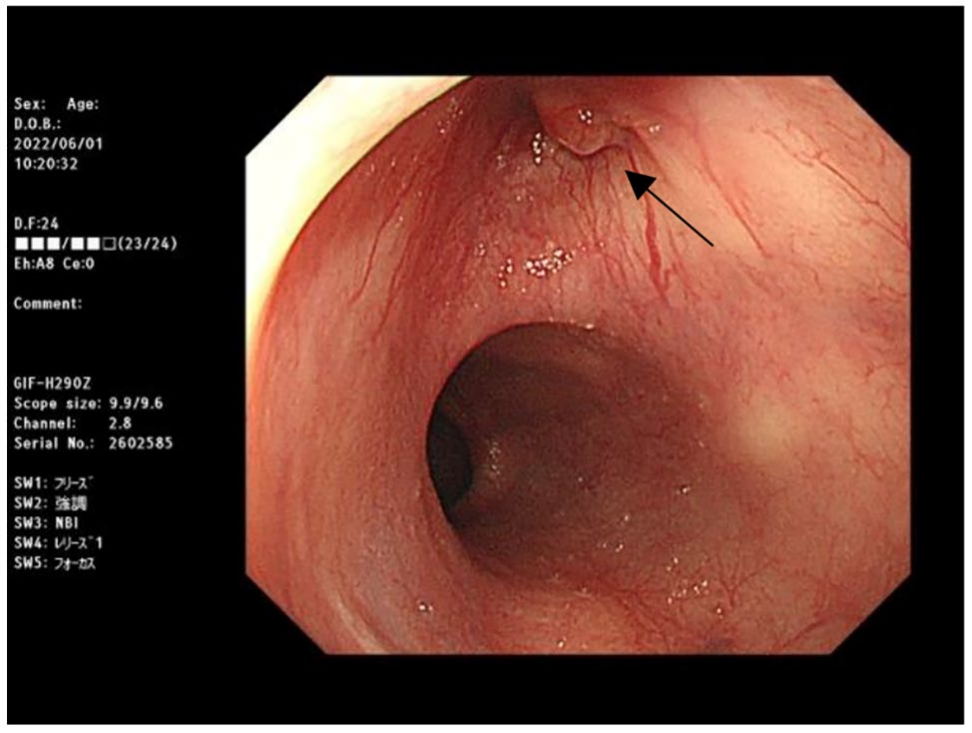
Esophagogastroduodenoscopy, black arrow indicates an erosion site at 25 cm below the incisor

## Operative procedure

The patient was placed in the right decubitus position. Left posterolateral thoracotomy was performed, and we located the sixth intercostal space without rib cutting method. The left common femoral artery and vein were prepared as well for cardiopulmonary bypass (CPB). After full-dose heparin was administered, we commenced CPB via the left common femoral vein, with the main pulmonary artery for venous drainage and the left common femoral artery for arterial perfusion. While the systemic body temperature was being lowered to 20 °C, we ligated the intercostal arteries as far as possible to identify the AEF site (Fig. 7). After the systemic body temperature had decreased to 20.6 °C, we clamped the mid-thoracic aorta for distal visceral artery perfusion and opened the aneurysm sac. For cerebral perfusion, we maintained the patient in the transverse Trendelenburg position and started retrograde cerebral perfusion via the left common femoral vein drainage. The previous stent graft was removed easily, and a balloon catheter was placed at the proximal aortic site to start antegrade cardioplegia. After dedicate debridement of the previous graft site, a 26-mm graft (Triplex, Vascutek Terumo, Tokyo, Japan) was continuously anastomosed to the previous surgical graft (24-mm four-branch J graft SHIELD NEO) with 3 − 0 Prolene. We clamped at the 26-mm graft after meticulous hemostasis and inserted another cannula from the 8-mm side branch of the graft for upper body perfusion. Thereafter, we started to rewarm the body. We clamped the left femoral arterial cannula and released the mid-thoracic aortic clamp for distal thoracic aorta open anastomosis while ligating the intercostal arteries. Distal aortic fenestration was created for chronic aortic dissection. The distal anastomotic site was thoracic aorta above the diaphragm. We anastomosed the other end of the 26-mm graft to the distal thoracic aorta with 4 − 0 prolene running suture and reinforced with 4 − 0 multiple double-pledgetted sutures. Deairing and declamping were performed after anastomosis (Fig. 8). After the systemic body temperature had returned to 36 °C, the CPB was weaned off and hemostasis was monitored.

**Fig. 6:**
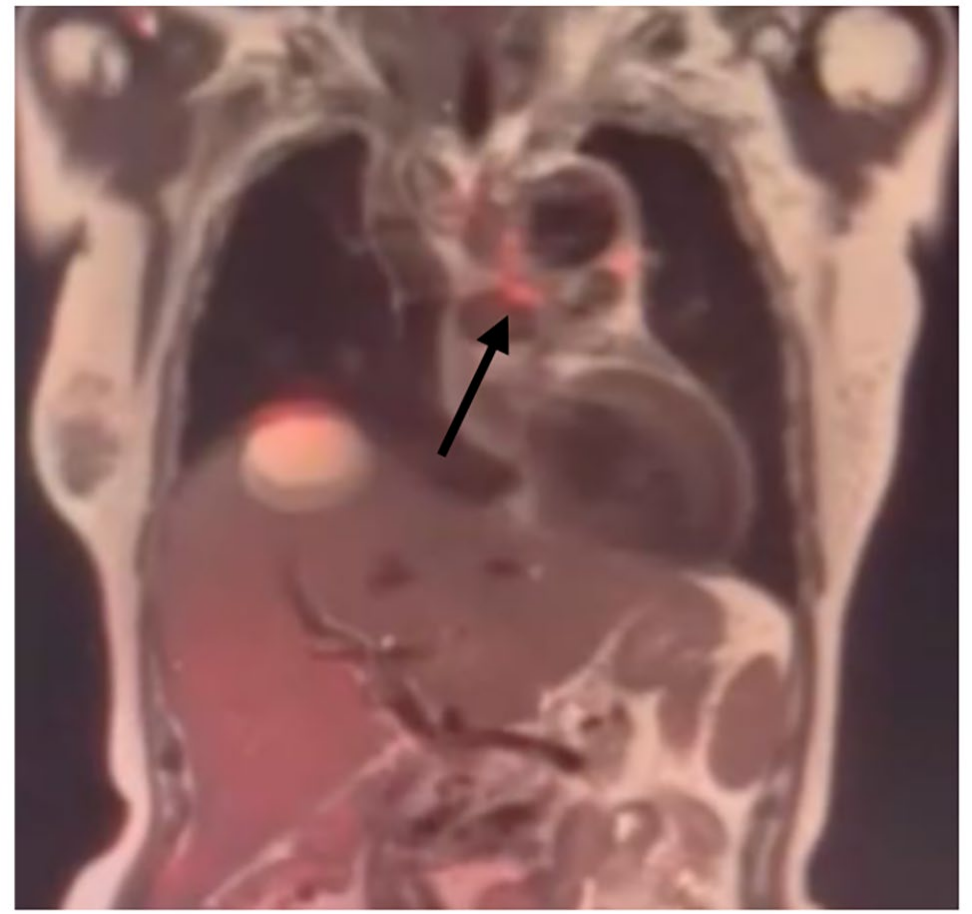
PET-MRI scan and the black arrow showed aortoesophageal fistula formation and the extent of infection

**Fig. 7:**
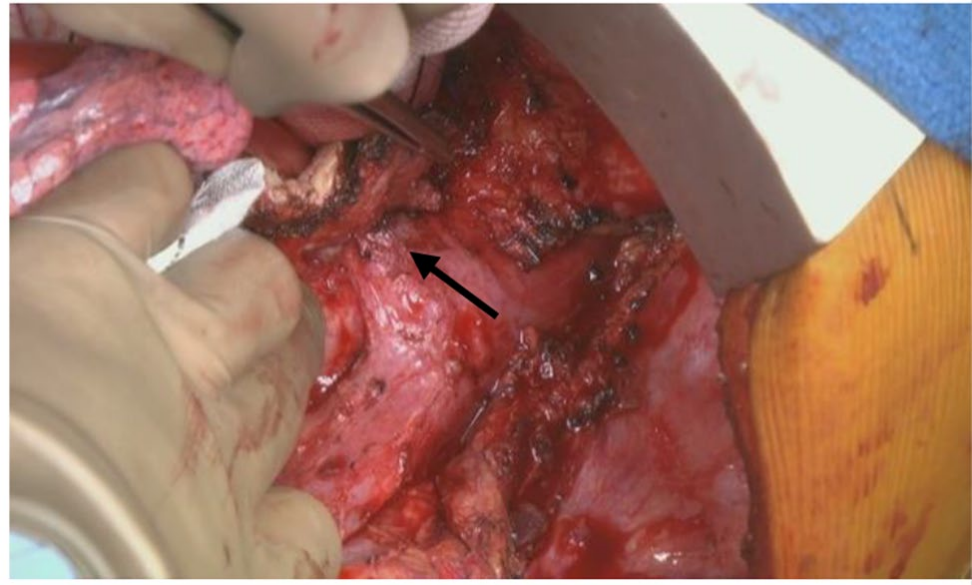
Black arrow is the aortoesophageal fistula site, which shows the esophagus directly adhesive on the aorta

Total esophagectomy, gastric tube reconstruction, and omental flap were performed by gastrointestinal surgeons. Gastric tube reconstruction was performed via the posterior mediastinal route (Fig. 9). After meticulous hemostasis, the wound was closed in layers.

**Fig. 8:**
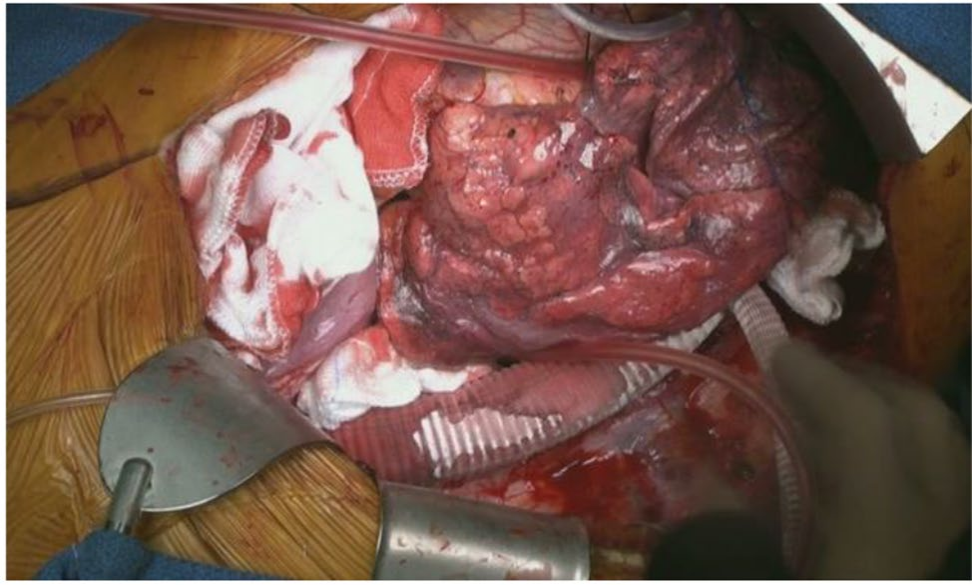
After thoracic aortic replacement

## Clinical process

After surgery, the patient was sent to intensive care unit for observation. She was extubated the next day and transferred to the general ward on postoperative day 3. On postoperative days 7 and 14, we followed up with CT scan, which demonstrated favorable hemostasis and no residual air pocket accumulation around the graft site. The patient tolerated the whole process well, and antibiotic treatment was maintained. Her CRP level also decreased (26.2 mg/L to 1.2 mg/L). Her general condition was stable, and she was discharged after a 6-week antibiotic course.

**Fig. 9:**
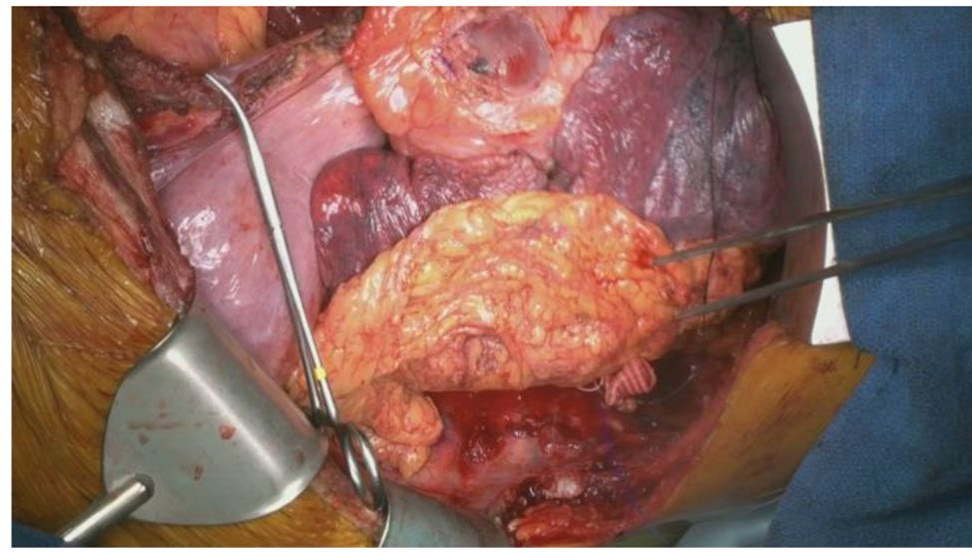
After gastric tube reconstruction and omental flap coverage

## Discussion and conclusions

AEF secondary to thoracic aortic aneurysm repair is a rare and fatal condition [1]. Furthermore, the optimal treatment for AEF remains controversial between intraluminal stent graft and open surgery, including extra-anatomical bypass and thoracic aortic replacement [2–5].

Stent graft for the treatment of AEF has been used especially in patients with acute aortic syndrome, but without follow-up treatment, the long-term mortality rate remains high without subsequent therapy. Conservative treatment is not a viable option according to other report [6]. Definite aortic treatment and esophageal reconstruction is crucial to prevent secondary infection of the aortic prosthesis but is usually accompanied by high morbidity and mortality.

Several case series have discussed the feasibility of extra-anatomical bypass to prevent secondary infection and local debridement. However, some reports have reported risking lower body malperfusion by bilateral axillofemoral bypass [3].

The omental flap has been used in deep sternal infection such as post-operative mediastinitis for several decades. Some case reports have discussed using omental flap to treat aortoesophageal fistula. Most of them were treated with aortoesophageal fistula directly repaired and omental flap was harvested thereafter to cover between aortic stent graft and esophagus. And this gave us ideas about using omental flap after total reconstruction surgery to. In our opinion, we thought that total reconstruction surgery remained the gold standard treatment for active in.

Our standardized protocol was as follows: (1) infected stent graft removal followed by thoracic aortic replacement; (2) total esophagectomy and gastric tube reconstruction; and (3) omental flap to cover the new aortic graft. We suggest that young patients whose ECOG performance status scale is less than two may need further aggressive treatments. We believe that the omental flap to cover the new aortic graft that has the advantage that can be isolated from primary infection site.

For aortic graft replacement, we chose left posterolateral thoracotomy approach using CPB under deep hypothermia circulatory arrest (DHCA) to prevent proximal aortic clamping. According to our previous thoracoabdominal aortic replacement result, DHCA demonstrates favorable protective effect on the spinal cord, with low stroke rate [7]. Extra-anatomical bypass is not considered for cases involving high morbidity and risk of further aortic rupture. After protamine reversal, meticulous hemostasis was performed for the next esophageal reconstruction surgery. Through the same wound, we freed the esophagus from the cervical esophagus to esophagogastric junction. Thereafter, we consulted a general surgeon for further gastric tube reconstruction and omental flap coverage via the posterior mediastinal approach.

In this case, the patient underwent TEVAR surgery for AEF, but the efficacy remains unclear. We suggested direct total reconstruction, including thoracic aortic replacement and esophageal reconstruction after 2 weeks of antibiotic treatment. In AEF cases presenting with acute aortic syndrome, TEVAR stent is a viable option, but we suggest that all patients need to undergo second-stage operation. Herein, we reported a case of postoperative AEF treated using one-stage total repair, including (1) stent graft removal and thoracic aortic graft replacement, (2) total esophagectomy, (3) esophagus reconstruction with gastric tube, and (4) omental flap coverage of the aortic graft to prevent secondary infection. Due to effective results in our hospital, definite aortic treatment and debridement must be aggressive in these patients.

## Data Availability

The datasets used and/or analysed during the current study are available from the corresponding author on reasonable request.
